# Exploring the role of the Ser9Gly (rs6280) Dopamine D3 receptor polymorphism in nicotine reinforcement and cue-elicited craving

**DOI:** 10.1038/s41598-020-60940-4

**Published:** 2020-03-05

**Authors:** Chidera C. Chukwueke, William J. Kowalczyk, Patricia Di Ciano, Marie Gendy, Richard Taylor, Stephen J. Heishman, Bernard Le Foll

**Affiliations:** 1 Translational Addiction Research Laboratory, Centre for Addiction and Mental Health (CAMH), Toronto, ON Canada; 2 National Institute on Drug Abuse, Intramural Research Program, Baltimore, United States of America; 3 Department of Pharmacology and Toxicology, University of Toronto, Toronto, Ontario Canada; 4 Acute Care Program, CAMH, Toronto, ON Canada; 5 CAMH, Campbell Family Mental Health Research Institute, Toronto, ON Canada; 6 Department of Family and Community Medicine, University of Toronto, Toronto, ON Canada; 7 Division of Brain and Therapeutics, Department of Psychiatry, University of Toronto, Toronto, ON Canada; 8 Institute of Medical Sciences, University of Toronto, Toronto, ON Canada; 9 Institute for Mental Health Policy Research, Centre for Addiction and Mental Health, Toronto, ON Canada; 10 Department of Psychology, Hartwick College, Oneonta, New York, United States of America

**Keywords:** Clinical pharmacology, Behavioural genetics

## Abstract

Preclinical studies show that the dopamine D3 receptor (D3R) is involved in the reinstatement of drug seeking and motivation for drugs of abuse. A D3R gene variant, Ser9Gly (rs6280) has been linked to nicotine dependence, yet the mechanisms underlying its involvement in nicotine dependence is unclear. This study investigated the relationship between the Ser9Gly variant and measures of both nicotine reinforcement and cue-elicited craving. Phenotypes of smoking behaviors were assessed in genetically grouped (Glycine vs. No Glycine carriers) current smokers (n = 104, ≥ 10 cigarettes per day). Laboratory measures included a forced choice session (to measure reinforcement of nicotine containing vs. denicotinized cigarettes), and a cue-reactivity session (to measure smoking cues vs. neutral cues elicited craving). The forced choice procedure revealed that subjective ratings were significantly higher in response to nicotinized compared to denicotinized cigarettes; however the Ser9Gly variant did not influence this effect. By comparison, smoking cues elicited greater craving over time compared to neutral cues, and Glycine carriers of the Ser9Gly D3R variant seem to experience a significant blunted cue-elicited craving effect. Results support D3R involvement in nicotine cue reactivity. However, more research is needed to reveal how this gene variant modulates various aspects of nicotine dependence.

## Introduction

In 2017, 14% of the U.S. population were current cigarette smokers, which, in combination with smoking being the leading cause of preventable death, present a significant health burden in the country^1^. However, while two-thirds of adult smokers want to quit smoking, less than one-third achieve this goal, suggesting suboptimal smoking cessation therapies^2^. One way to address this deficiency is to take advantage of genetic variation to personalize treatments^3^. Given the importance of dopaminergic system in addictive processes^4,5^, the genes associated with dopamine receptors have gained considerable research attention^6^.

Recently, the dopamine D3 receptor subtype (D3R) has been implicated in nicotine-related reinforcement and craving^7^. Preclinical models of nicotine reward have shown that selective antagonism of the D3R attenuates nicotine self-administration under a progressive ratio schedule^8^ and the expression of nicotine induced conditioned place preference^9,10^. Animal models of craving have also show that selective antagonism of D3R attenuated cue- and drug-induced reinstatement of nicotine seeking in rats^11,12^.

The Ser9Gly single nucleotide polymorphism (SNP; rs6280) results in D3R variants that have been identified and linked to smoking behavior in humans. The Ser9Gly D3R SNP corresponds to a serine to glycine substitution at position 9 of the N-terminal extracellular domain. The serine allele (T allele) is the major allele present in 51% of the global population, while the minor Glycine allele (C allele) is present in 49% of the population^13^. *In vitro* studies using binding assay techniques have observed that homozygotic Glycine alleles yield D3R receptors that have increased affinity for dopamine^14,15^. In a study done by Lundstrom and Turpin^14^, CHO cells were infected with a virus resulting in high level expression of recombinant receptors (either wildtype, homozygous, or a combination of the two). Homozygotic glycine allele receptors (pK_i_ values for dopamine; 8.16 ± 0.38) had significantly higher affinity for dopamine compared to glycine heterozygotes (7.34 ± 0.16) and serine homozygotes (7.38 ± 0.23). Additionally, in Jeanneteau *et al*.^15^, HEK293 cells transfected with the two DRD3 variants were used to assess the ability of dopamine to inhibit the binding to D3R in homozygotic Glycine versus Serine D3Rs. This study reported that the Ser9Gly mutation increased dopamine affinity by 4–5 times^15^. Because these studies were performed in artificial cellular systems, the results need to be replicated and may not be fully reflective of the human situation. However, with those caveats in mind, the data suggests that in glycine carriers, D3Rs may have enhanced affinity for dopamine.

A human genetic study investigating 13 D3R-related SNPs showed that the Ser9Gly polymorphism was positively associated with various measures of nicotine dependence (i.e., Smoking quantity, Heaviness of Smoking Index (HSI), and the Fagerstrom test for nicotine dependence (FTND)) in European-American smokers^16^. In another study, the Glycine allele of the Ser9Gly variant was associated with increased smoking, indicated by lower time to first cigarette from waking and increased values of the HSI^17^. However, while the literature demonstrates an association between the Ser9Gly polymorphism and smoking-related self-report measures, no study has investigated the influence of this polymorphism on laboratory measures of smoking behavior.

The current study investigated the relationship between the Ser9Gly polymorphism and human laboratory measures of smoking behavior. In particular, this study explored the association of the Ser9Gly variant with nicotine reinforcement and with cue-elicited craving as measured by a forced choice and cue-exposure procedure, respectively. Research has previously demonstrated nicotine reinforcement^18–20^ and smoking cue-elicited craving^21–23^ using similar methods. We hypothesized that our findings would align with previous literature and that the glycine mutation for D3R would be associated with greater choices for Nicotinized (Nic) cigarettes puffs, compared to Denicotinized (Denic) puffs, and greater reactivity to smoking cues, compared to neutral cues. Additionally, due to previous literature pointing to decreased smoking related reward^24^ and greater sensitivity to smoking cues^25^ in female smokers, we hypothesize that sex may similarly affect our measures of nicotine reinforcement and cue-reactivity.

## Methods

### Participants

The data reported here was pooled from three different studies conducted at two different sites. One study investigated the relative reinforcing efficacy of nicotine, the effects of environmental cues on autonomic responsivity and potential associations between genetic polymorphisms and both of those phenotypic measures (Protocol #10-DA-N456). This study was conducted in Baltimore, Maryland (n = 55). The second study, conducted in Toronto, Ontario (n = 23), investigated the effects of gemfibrozil on the same measures^26^. This report only includes data from the placebo condition of the study to avoid confounding effects of the medication. The final study, also conducted in Toronto, Ontario (n = 25), increased the sample size of participants on the same measures (REB# 134/2015). All studies were conducted according to the Declaration of Helsinki (7^th^ revision) as well as protocols approved by the respective review boards of the National Institute of Drug Abuse (NIDA) and the Centre for Addiction and Mental Health (CAMH). Subjects were eligible if they were 18–64 years old, smoked at least 10 cigarettes per day (CPD) for at least one year, had positive urinary cotinine levels (for smoking confirmation), and were medically and psychologically healthy. To determine overall health, medical and psychiatric history was collected in all studies. Subjects were ineligible if they were seeking treatment for nicotine dependence, recently used nicotine replacement products, consumed more than 15 alcohol drinks per week, used illicit drugs regularly, were pregnant or nursing, or used medications that would be unsafe during experimental sessions.

### Study design

Participants enrolled in the various studies first attended an in-person eligibility assessment, where, informed consent was obtained followed by collection of demographic and drug use related data. Data collected during this session included breath samples for breath alcohol concentration (BrAC) and carbon monoxide (CO) estimations using breathalyzers. This information was used to verify negative drinking and positive smoking status. A urine drug screen was performed and females underwent a urine pregnancy test. Vital signs were collected during this session and subjects completed questionnaires including the FTND^27^. Those who met inclusion/exclusion criteria were subsequently enrolled, invited to provide blood for genotyping at a convenient time during the study, and participate in the forced-choice and cue reactivity experimental sessions. Both experiments were conducted in a room that contained a 2-way mirror, through which research observations could be made. The forced-choice occurred first and on a separate day than the cue reactivity session for all studies.

### Questionnaires

The modified Cigarette Evaluation Questionnaire (mCEQ)^28^ was used to measure the subjective effects of cigarettes during the forced choice session. During the cue-reactivity session, craving was assessed by Tobacco Craving Questionnaire – Short Form (TCQ-SF)^29^ and the Visual Analogue Scale (VAS)^30^. Using the VAS, participants were asked to rate how much they ‘craved’ and ‘urged’ for a cigarette and that specific moment. Mood was also measured during the cue-reactivity session using the Mood Form^31^, and the VAS, where they were asked about ‘positive’ and ‘negative’ mood at that specific moment.

### Forced-choice procedure

This double-blinded procedure examined the relative reinforcing effects of nicotine^32,33^. There were 3 phases during this procedure; smoking deprivation, cigarette exposure trials, and the force-choice trials. First, participants were seated in a specialized ventilated room and were instructed to smoke 4 puffs of their own cigarette. Subsequently, CO was measured and they relaxed in the room where they read or listened to music, without smoking, for 30–60 minutes. This standardized the time since last cigarette. After this smoking deprivation period, participants began the cigarette exposure phase.

#### Cigarette exposure

Participants sampled research cigarettes that differed in amounts of nicotine yield. Nic cigarettes contained commercially available amounts of nicotine, while Denic cigarettes contained negligible amounts of nicotine (see ‘cigarettes’ section). Participants underwent 4 exposure trials where they sampled either Nic (A) or Denic (B) color-coded cigarettes in an ABAB or BABA order counterbalanced across all participants. Exposure trials were completed in 20–30 minute intervals to avoid nicotine satiation and simulate regular smoking behavior (8 puffs every 40 minutes^34^). After each exposure trial, participants completed the mCEQ. These mCEQ scores were averaged across both Nic (A) or Denic (B) trials.

#### Forced-choice trials

20–30 minutes after the last exposure trial, participants completed 4 forced choice trials in 20–30 minute intervals. Here, participants were concurrently presented both Nic and Denic cigarettes, and instructed to smoke any combination of a total of 4 puffs from either or both cigarettes. The participant’s choice was recorded by observation through a 2-way mirror.

#### Cigarettes

The cigarettes used in the various studies differed slightly due to manufacturing and accessibility constraints. The study conducted in Baltimore (Protocol #10-DA-N456) used Quest® 1 cigarettes (Vector Tobacco), which contained 0.6 mg nicotine (Nic), and Quest® 3 cigarettes, which contained less than 0.05 mg nicotine (Denic). The study investigating gemfibrozil’s effect on nicotine dependence^26^ used commercially available Player’s Rich brand cigarettes (Nic) and the same Quest 3® cigarettes (Denic). The most recent study intended to increase sample size (REB# 34/2015) used SPECTRUM® research cigarettes (RTI international) that contain 0.9 mg nicotine (Nic) and 0.03 mg nicotine (Denic).

### Cue-reactivity

Participants underwent a smoking deprivation period where they smoked 4 puffs of their own cigarette, then relaxed for 30–60 minutes. After the last puff, a CO sample was taken and the participants completed baseline self-report measures (TCQ-SF, VAS, and Mood Form). The cue exposure began after the deprivation period and participants were connected to a physiological recording device (Biopac device model: #INI14020000901). In this phase, subjects were presented an opaque container containing either a smoking-related or neutral cue. Participants were instructed to lift the cover of the container and interact with the objects inside. In the smoking cue condition, the container housed a standard pack of commercially available cigarettes, a lighter, and an ash tray. Participants were told to light the cigarette without puffing and hold the cigarette for 30–60 seconds, after which it was extinguished. In the neutral cue condition, the cue container housed a pack of pencils, a sharpener, and a notepad. Participants were instructed to take a pencil, sharpen it, and hold it as if writing for 30–60 seconds. Participants completed the TCQ-SF, VAS, and Mood Form prior to cue exposure (baseline) and 15-minutes after cue exposure. There was one exposure session for each cue type (neutral and smoking) and the order of cue presentation (smoking or neutral first) was counter-balanced across all participants. Physiological readings (heart rate, blood pressure, skin conductance and skin temperature) were collected throughout the session.

### Genotyping

Genotyping for all data sets were done at CAMH. Approximately 20 ml of blood was collected during one of the experimental visits. DNA was extracted from whole blood in ethylenediaminetetraacetic acid (EDTA). Total genomic DNA was genotyped using commercially available TaqMan SNP genotyping assays as per the manufacturers’ directions for rs6280 (assay ID C____949770_10).

### Data analysis

The data set was split across gene variants (Glycine carriers vs. No Glycine carriers) and analyses were conducted to explore the role of the Ser9Gly variant in the forced choice and cue-reactivity paradigms. For the forced choice session, subjective responses to cigarette types were evaluated using the mCEQ. Analysis included both the mCEQ composite score^35^ and subscales (Psychological Reward, Aversion, Craving Reduction, Respiratory Tract Sensation, and Smoking Satisfaction). The behavioral response to the forced choice examined number of puff choices on Nic versus Denic cigarettes (out of a maximum of 16 possible choices). All variables were analyzed using a mixed model repeated-measures cigarette type x sex x gene variant ANOVAs where cigarette type (Nic, Denic) was a within-subject variable, while sex (male, female) and gene variant (Glycine, No Glycine) were between-subjects variables.

Cue-reactivity outcomes were collected at two time points (baseline and 15 minutes after cue). Values over time were calculated as difference scores (15 minutes after cue minus baseline). Difference scores of subjective (TCQ-SF [general and individual factors], VAS [craving and mood], Mood Form) and physiological (skin conductance, skin temperature, heart rate, and blood pressure) data were created. These variables were analyzed using a mixed model repeated-measures cue type x sex x gene variant ANOVAs where cigarette type (Nic, Denic) was a within-subject variable, while sex (male, female) and gene variant (Glycine, No Glycine) were between-subjects variables. In the results, we describe the main effects for the cue reactivity and forced choice ANOVAs in the first section, and the outcomes of the interactions in the second.

Separate analyses were run to explore the potential effects of race and cigarette brand. For these analyses the same dependent variables were used to examine the forced choice and cue reactivity. To examine the effects of race (Whites, Blacks, and others), race × cigarette (or cue) type ANOVAs were run. To examine the effect of Denic cigarette brand (Spectrum, Quest), cigarette type × cigarette brand ANOVAs were run. For all analyses, missing data was substituted with means, and results were considered significant at p < 0.05 (SPSS ver. 24.0/25.0). Bonferroni’s corrections were applied on all multiple comparisons.

## Results

### Participants

A total of 104 participants were recruited, genotyped, and completed all components of the study (See Table 1). There were no significant differences between genotype groups across all demographic and smoking characteristics, except for race where there was a higher concentration of white people in the No Glycine group compared to a higher concentration of black people in the Glycine group. Data analysis on forced choice data included the entire data set (n = 104) while analysis on cue-reactivity (n = 103) excluded one participant due to lack of questionnaire data.

**Table 1:** .

		Total N = 104	Glycine n = 62	No Glycine n = 42	2-tailed t test and χ^2^ analysis (Glycine vs. No Glycine)
Demographic Characteristics		n (%)	n (%)	n (%)	p
Sex	Male Female	57 (54.8) 47 (45.2)	36 (58.1) 26 (41.9)	21 (50.0) 21 (50.0)	n.s.
Race	Black White/Caucasian Other	31 (29.8) 63 (60.6) 10 (9.6)	27 (43.5) 31 (50.0) 4 (6.5)	4 (9.5) 32 (76.2) 6 (14.3)	0.001
Education	> high school	63 (60.6)	33 (54.1)	30 (71.4)	n.s
		M ± SD	M ± SD	M ± SD	p
Age (years)		41.80 ± 11.07	40.69± 11.16	43.43 ± 10.86	n.s.
Smoking Characteristics					
Cigarettes per day (CPD)		17.48 ± 5.85	17.4 ± 5.82	17.6 ± 5.97	n.s.
# of smoking years		22.27 ± 11.43	20.94 ± 11.01	24.25 ± 11.88	n.s.
Fagerstrom Test for Nicotine Dependence (FTND; n = 103)		5.35 ± 1.89	5.30 ± 1.86	5.43 ± 1.97	n.s.

This table describes the demographic and smoking characteristics of the study sample. The sample was compared across genetic groups using χ^2^ analysis for categorical variables and 2-tailed t tests for continuous variables. n.s. = not significant.

### Main effects of nicotine and smoking cues

#### Forced choice

**Figure 1:**
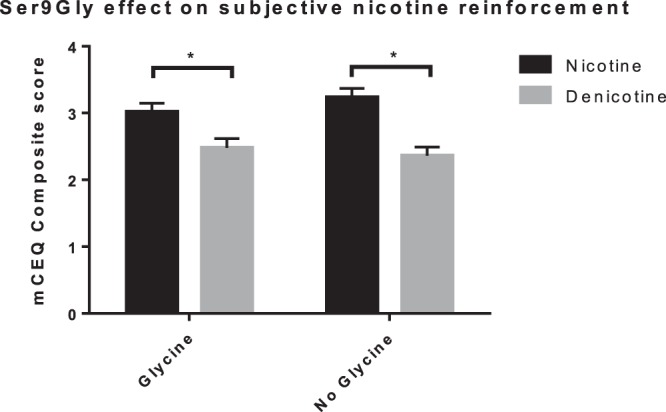
mCEQ composite score. mCEQ composite score for Nic and Denic cigarettes across both genetic groups. There was a main effect of cigarette type (F (1,100) = 50.911, p < 0.001, η_P_^2^ = 0.337) and no gene related interaction effects. In both genetic groups, Nic cigarettes were rated significantly higher than Denic cigarettes. *Nic* = Nicotinized, *Denic* = Denicotinized, *mCEQ* = Modified Cigarette Evaluation Questionnaire.

There was a significant main effect of cigarette type in the mCEQ composite score (Fig. 1; F (1,100) = 50.911, p < 0.001, η_P_^2^ = 0.337) as well as all mCEQ subscales (‘craving reduction’ (F (1,100) = 49.263, p < 0.001, η_P_^2^ = 0.330); ‘enjoyment of the respiratory tract sensation’ (F (1,100) = 27.490, p < 0.001, η_P_^2^ = 0.216); ‘smoking satisfaction’ (F (1,100) = 52.070, p < 0.001, η_P_^2^ = 0.342); ‘psychological reward’ (F (1,100) = 26.826, p < 0.001, η_P_^2^ = 0.212) with the exception of the ‘aversion’ (p > 0.05). The mCEQ composite scores (mean ± standard deviation; M ± SD) show that Nic cigarettes (3.11 ± 0.96) were rated higher than Denic cigarettes (2.43 ± 1). This was similar in all subscales except aversion, where both cigarette types elicited comparable scores, suggesting that Nic cigarettes were rated higher than Denic cigarettes on positive aspects of smoking. Findings were not affected by cigarette brand (Spectrum vs. Quest).

**Figure 2:**
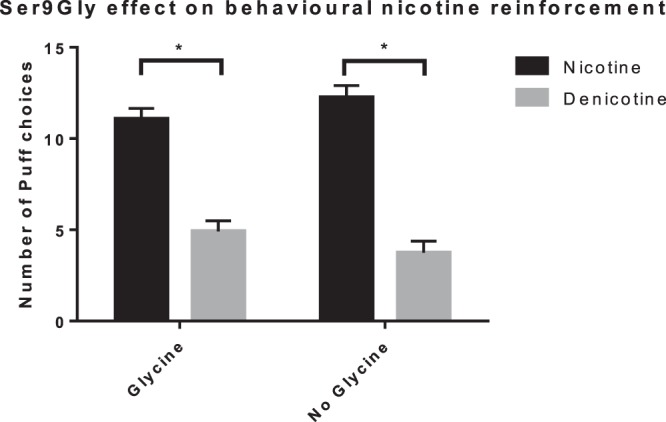
Forced choice. Number of puff choices from Nic and Denic cigarettes across both genetic groups in the forced choice task. There was a main effect of cigarette type, where participants chose puffs (mean ± standard deviation) of the Nic cigarette (11.56 ± 4.42) significantly more than the Denic cigarette (4.44 ± 4.42; F (1,100) = 67.410, p < 0.001, η_P_^2^ = 0.403) in both genetic groups. *Nic* = Nicotinized, *Denic* = Denicotinized.

In the forced choice trial, there was a main effect of cigarette type, suggesting that participants chose puffs (M ± SD) of the Nic cigarette (11.56 ± 4.42) significantly more than the Denic cigarette (4.44 ± 4.42) (Fig. 2; F (1,100) = 67.410, p < 0.001, η_P_^2^ = 0.403). Taken together with the subjective measures, these results show that Nic cigarette elicited higher positive subjective ratings as well as puff choices compared to Denic cigarettes. Puff choice was not affected by cigarette brand.

#### Cue-reactivity

**Figure 3:**
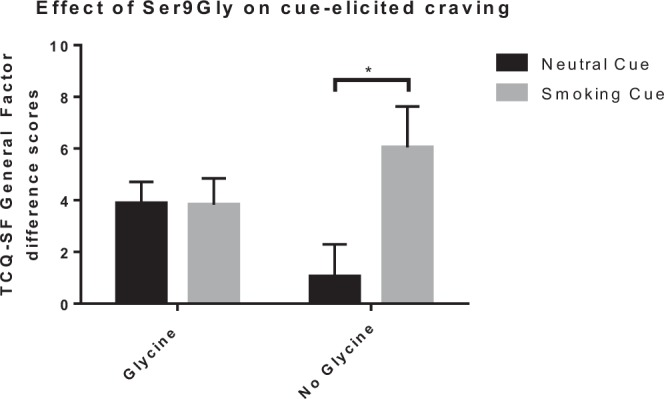
Craving. Craving, assessed by the TCQ-SF General Factor, elicited by smoking and neutral cues for both genetic groups. There was a cue type × gene variant interaction (F (1,99) = 4.218, p = 0.043, η_P_^2^ = 0.041), and bonferroni-corrected multiple comparisons showed that, for the No Glycine group, but not the Glycine group, difference scores were significantly higher in the smoking cue condition compared to the neutral condition (adjusted p = 0.005). *TCQ-SF* = Tobacco Craving Questionnaire – Short Form.

There was a significant main effects of cue type on all craving measures (the VAS-crave scale [F (1,99) = 4.859, p = 0.030, η_P_^2^ = 0.047]; the VAS-urge scale [F (1,99) = 4.475, p = 0.037, η_P_^2^ = 0.043]; the TCQ-SF general factor [Fig. 3; F (1,99) = 4.218, p = 0.043, η_P_^2^ = 0.041]). For all 3 measures, mean (± SD) craving difference scores were higher in the smoking cue condition compared to the neutral cue condition (VAS-crave: 11.2 ± 17.63 vs 5.48 ± 19.49; VAS-Urge: 10.59 ± 17.41 vs 5.38 ± 18.56; TCQ-SF general factor: 4.74 ± 8.95 vs 2.73 ± 7.30). This suggests that smoking cues generally elicited greater craving over time compared to neutral cues.

#### Mood

There was a main cue type effects on negative mood (F (1,99) = 6.664, p = 0.011, η_P_^2^ = 0.063) and a trend-level cue type effect on positive mood (F (1,99) = 3.662, p = 0.059, η_P_^2^ = 0.036), as assessed by the Mood Form. Analyses of difference scores (M ± SD) revealed a greater increase in negative mood when presented with a smoking-related cue (1.02 ± 2.86) compared to a neutral cue (0.14 ± 3.23). The weaker cue type effect on positive mood may suggest less influence of smoking related environmental cues on positive compared to negative mood. These effects were not seen in mood assessed by the VAS, suggesting a decreased sensitivity to cue-elicited mood changes compared to the Mood Form. Overall, these results indicate greater smoking cue-elicited elevation in negative mood over time, as assessed by the Mood Form.

#### Physiological measures

Data was analyzed from 78 participants; the remaining data was not analyzed due to technical issues. Analyses on physiological measures showed a main cue type effect on heart rate (F (1,57) = 4.556, p = 0.037, η_P_^2^ = 0.074). Here, smoking related cues (M ± SD) elicited greater decreases (−2.03 ± 5.10) in heart rate compared to neutral cues (−0.41 ± 3.5). There were no main effects of cue on any of the remaining physiological measures (skin conductance, blood pressure, and skin temperature; data not shown).

### Genotype interactions

#### Forced choice

There were no significant gene interaction effects on either mCEQ composite score or the forced choice task (Figs. 1,2). These results indicate that, while Nic cigarettes resulted in higher subjective ratings and more puff choices than Denic cigarettes, this effect is similar in both genetic groups, suggesting a lack of Ser9Gly effect on nicotine reinforcement.

#### Cue-reactivity

There was a cue type × gene variant interaction in the TCQ-SF General Factor (Fig. 3; F (1,99) = 4.218, p = 0.043, η_P_^2^ = 0.041), suggesting that craving elicited by the different cue conditions depended on the genetic group. Bonferroni-corrected multiple comparisons showed that, for the No Glycine group, but not the Glycine group, difference scores were significantly higher in the smoking cue condition compared to the neutral condition (adjusted p= 0.005). There were no interaction observed in the VAS-Crave and -Urge scales (ps < 0.05). This finding was not affected by race.

Analyses of the TCQ-SF factors 1–4 difference scores showed a significant cue type × gene variant interaction only in TCQ-SF Factor 4 (F(1,99) = 8.839, p = 0.004, η_P_^2^ = 0.082). Bonferroni’s multiple comparisons revealed that non-Glycine carriers, but not Glycine carriers, had greater increases in smoking cue-elicited TCQ-SF Factor 4 (‘purposefulness’) responses compared to the neutral cue (adjusted p = 0.004).

#### Mood

There were no gene interaction effects on mood changes in response to the different cue conditions as assessed by the Mood Form and VAS mood scales.

#### Physiological measures

Mixed model (cue type × sex × gene variant) ANOVAs showed no gene related interaction effects.

### Supplemental analyses

The brand of Denic cigarettes used in the study did not affect subjective or behavioural measures of nicotine reinforcement (data not shown).

Race did differ in mCEQ ratings (cigarette type × race: F (1,101) = 3.889, p = 0.024, η_P_^2^ = 0.071), with the cigarette type effects seen in all races except for black participants. Race also differed in the forced choice task (cigarette type × race: F (1,101) = 5.341, p = 0.006, η_P_^2^ = 0.096) with a greater cigarette type effect seen in white participants compared to black participants (adjusted p = 0.005). Finally, race affected positive mood assessed by the Mood Form (F (1,100) = 4.526, p = 0.013, η_P_^2^ = 0.083), with black participants experiencing greater cue effects compared to other races. There were no other effects of race.

## Discussion

This study found that Nic, compared to Denic cigarettes, elicited greater positive responses on the mCEQ and puffs choices in the forced choice task, however this was not influenced by the Ser9Gly gene variant. In the cue-reactivity paradigm, smoking cues elicited greater craving than neutral cues, and this effect may have been blunted in smokers with the Glycine allele of the Ser9Gly SNP. While these results suggest nicotine-induced reinforcement and craving, our analysis indicates that the Ser9Gly may be more influential in smoking-cue-related craving rather than nicotine reinforcement, which is in partial alignment with our hypothesis. Overall, there were no significant effects of sex in our analysis, which was inconsistent with our hypothesis.

Our data suggests that nicotine reinforcement was not affected by the Ser9Gly D3R polymorphism as measured by the mCEQ and the forced choice task. The finding of differential subjective ratings in the present study is consistent with previous studies that show increased subjective responses to Nic cigarettes compared to Denic cigarettes^18,35,36^. This supports the notion that positive subjective effects of smoking may be attributable to nicotine itself. Consistent with previous studies^18–20^, this study also found that smokers given a concurrent choice between Nic and Denic cigarettes chose significantly more Nic puffs than Denic puffs, further suggesting that nicotine contributes to the behavioral reinforcement of smoking. Both Glycine and non-Glycine allele carriers of the Ser9Gly SNP produced higher subjective ratings in response to Nic cigarettes compared to Denic cigarettes. Results were similar in the forced choice task, where both genetic groups chose significantly more Nic puffs than Denic puffs. Together, this suggests that the Ser9Gly polymorphism may not have a significant effect on nicotine reinforcement as assessed by these subjective and behavioral measures.

While the link between nicotine reinforcement and dopamine transmission has been well characterized^37,38^, previous literature has shown that D3R antagonism does not affect the direct reinforcing effects of nicotine^12^ or cocaine^39,40^ under low self-administration requirements. Therefore, our findings in humans seem consistent with previous animal research, as the possible increased affinity for dopamine conferred by the D3R Ser9Gly mutation^14^ appears to have a minimal effect on human laboratory measures of nicotine reinforcement. On the contrary, our findings seem inconsistent with previous human studies that have associated the Ser9Gly SNP with measures of nicotine dependence^16^ and the Glycine allele with increased self-reported smoking behavior^17^. In light of the current findings, more research is required to better understand the relationship between the Ser9Gly SNP and nicotine reinforcement.

In the cue-reactivity session, smoking cues, compared to neutral cues, elicited greater craving over time, and the Ser9Gly polymorphism affected this finding. The finding of increased smoking cue-induced craving was statistically significant in all measures of craving in this study (TCQ-SF, VAS-Urge and -Crave). Previous studies have shown that subjects show greater craving in response to drug-related cues compared to neutral cues^41,42^ and this has been replicated using various modalities of smoking-related cues (i.e., virtual, visual, and olfactory) and various craving questionnaires^21–23^. Therefore, our findings are consistent with previously published reports.

Comparing across genetic groups, this study found that participants without the Glycine allele showed significantly greater smoking cue-induced craving compared to the neutral cue, and this was not seen in Glycine carriers. This suggests that the existence of the Ser9Gly SNP glycine allele (homozygotic or heterozygotic) produces a blunting effect on smoking cue-elicited craving over time. This study also found that this blunting effect of cue-elicited craving in the Glycine group was seen in the TCQ-SF subscale for ‘purposefulness’, suggesting that blunted cue-induced intention to smoke may be the driver for our observed effects.

The results from the cue-reactivity study are difficult to interpret in light of previous literature. Animal^11^ and human^43^ models have reported D3R-blockade-induced attenuation of nicotine craving, supporting an important role of D3R in nicotine craving. Our study also supports D3R involvement in nicotine craving, showing a blunted effect on smoking cue-elicited craving in those with an increased affinity for dopamine (i.e., Glycine carriers of the Ser9Gly SNP^14,15^). However, human studies have found a lack of association between dopamine release and measures of nicotine craving^42,44^ making it difficult to characterize the mechanisms which underlie our findings. Nevertheless, previous studies have shown genetic variations in responses to smoking-related cues^45–47^, and research has implicated the Ser9Gly SNP in nicotine dependence^16,17^. Therefore, the current study extends existing literature by supporting the involvement of the Ser9Gly SNP in nicotine craving, though more research is required to replicate this as well as elucidate the underlying mechanisms at play.

Our findings of mood changes in response to the different cues were inconsistent across the measures of mood (Mood form and VAS) but were unaffected by the Ser9Gly polymorphism. Our data showed significantly greater increases in negative mood and greater, though trend-level, decreases in positive mood and in response to smoking cues compared to neutral cues. This was found in the Mood form but not the VAS, suggesting greater sensitivity to mood changes in the Mood form. Indeed, some studies have shown smoking cue-induced changes in mood^25,48^, but not all^30,49^.

The current study showed greater smoking cue-induced decreases in heart rate compared to neutral cues, however this along with the other physiological measures, were not affected by the Ser9Gly SNP. The literature is mixed when considering heart rate responses to smoking cues, with some studies showing increased^50^, decreased^51^, and no heart rate changes^52^ in response to smoking cues. We did not find differences in skin conductance, inconsistent with other studies that have^53^. In total, our physiological measures were not as responsive to cue exposure as self-reports which has been seen in previous research^41,54^. Due to technical difficulties, our decreased sample size complicates interpreting these results.

All of the above observations should be considered within the context of some study limitations. First, because the data was collected from multiple studies, some study techniques were not completely consistent. For example, one study employed a 30-minute deprivation period, while another used a 60-miniute period. As craving has been shown to increase over time^55^, it is unclear how these inconsistent time periods affect our craving measures. Second, while our sample size is fair in comparison to other cue-reactivity studies, we may be underpowered to detect major effects when the sample is split across genetic groups (including heterozygous and homozygous groups). Finally, our recruitment sampling method did not consider race. Our sample included more black people with the Glycine allele compared to those without, similar to previous samples^16^. However, as race has been shown to be involved in the association between Ser9Gly SNP and nicotine dependence^16^ and because allelic frequencies may differ between ethnic groups^13^, we are unable to interpret how race affected our findings. Future research should include larger scale studies, consider race in population sampling, and use more consistent methods to address these limitations.

## Conclusion

In conclusion, study results from our human laboratory measure of nicotine reinforcement showed that nicotine elicited greater puff choices and higher positive subjective ratings. In addition, our cue reactivity paradigm showed that smoking related environmental cues elicited greater increases in craving compared to neutral cues. The Ser9Gly SNP had no effect in our measures of nicotine reinforcement but did influence cue-reactivity outcomes. Specifically, Ser9Gly Glycine carriers alone experienced a blunting effect in smoking cue-induced craving. This may suggest that the Ser9Gly SNP is more involved in nicotine related craving than reinforcement. More research is required to better understand the mechanistic role of the Ser9Gly D3R variant in nicotine craving.

## Data Availability

All additional data, research protocols, and information on materials used in this investigation will be made readily available upon request as allowed by the governing review boards of the involved research institutions.
